# Comparative performance of nine large language models on professional Parkinson’s disease tasks: a multidimensional benchmark study

**DOI:** 10.3389/fneur.2026.1970459

**Published:** 2026-10-30

**Authors:** Ludi Xie, Rumeng Wei, Li Xiong, Rui Lu, Kangwen Peng, Wenlin Ma

**Affiliations:** 1 Department of Geriatric Medicine, Tongji Hospital, School of Medicine, Tongji University, Shanghai, China; 2 Department of Neurology, Tongji Hospital, School of Medicine, Tongji University, Shanghai, China; 3 Center for Translational Neurodegeneration and Regenerative Therapy, Tongji Hospital Affiliated to Tongji University School of Medicine, Shanghai, China

**Keywords:** benchmarking, large language models, medical information systems, multidimensional evaluation, Parkinson disease

## Abstract

**Background:**

Parkinson’s disease (PD) requires both disease-specific knowledge and contextual clinical reasoning. However, most evaluations of medical large language models (LLMs) emphasize closed-ended questions and factual accuracy, providing limited insight into broader dimensions of performance on professional PD tasks.

**Methods:**

Nine contemporary LLMs were evaluated using a 200-item English-language professional PD benchmark spanning five clinical domains, four difficulty levels, and multiple question formats. Responses were assessed for expert-rated factual accuracy, clinical reasoning, Global Quality Scale (GQS), and Flesch–Kincaid Grade Level (FKGL).

**Results:**

The nine models generated 1,800 responses. Between-model separation was minimal for expert-rated factual accuracy (Kendall’ s W = 0.031) but greater for FKGL (W = 0.235), Clinical Reasoning (W = 0.256), and GQS (W = 0.301). Between-model separation in expert-rated factual accuracy was small for closed-ended questions (W = 0.020, *p* = 0.001) but greater for open-ended questions (W = 0.121, *p* < 0.001). Expert-rated factual accuracy remained weakly discriminative across PD domains and difficulty levels, while GQS showed the greatest domain-specific separation in Safety & Ethics (W = 0.431). Flagship variants consistently showed higher Clinical Reasoning and GQS, but not uniformly higher factual accuracy.

**Conclusion:**

Contemporary LLMs showed limited between-model separation in expert-rated factual accuracy within this benchmark and scoring framework, whereas greater differentiation emerged in open-ended tasks, clinical reasoning, overall response quality, and readability. Multidimensional evaluation may therefore provide a more informative characterization of model performance and help inform task-specific model selection and oversight in medical applications.

## Introduction

Parkinson’s disease (PD) requires not only factual knowledge but also the integration of symptoms, disease course, treatment response, and risk factors in diagnostic and therapeutic decision-making (1–4). Relevant professional tasks include differentiating idiopathic PD from atypical or secondary parkinsonian syndromes, as well as managing motor complications, non-motor symptoms, and device-aided therapies (2, 4). PD therefore provides a disease-specific setting that combines broad knowledge requirements with substantial reasoning demands, allowing assessment of whether large language models (LLMs) can translate medical knowledge into clinically contextualized responses.

Most existing evaluations of medical LLMs rely on standardized examinations or closed-ended questions, with final-answer accuracy as the primary outcome (5–9). Although such approaches are useful for assessing factual knowledge, they provide limited insight into whether models can present essential clinical information comprehensively, justify diagnostic or therapeutic choices, and appropriately qualify uncertainty and risk (6, 10–14). A model may reach the correct conclusion while omitting important differential diagnoses, contraindications, or alternative options; conversely, a fluent and detailed response may contain factual errors or unsupported recommendations (10–12, 15, 16). For medical students and clinicians, these “correct but incomplete” or “fluent but unreliable” responses may be particularly misleading, and accuracy-based evaluation alone may underestimate the risks associated with professional use (10, 12, 15, 16).

Evidence specific to PD remains limited. Previous studies have mainly evaluated ChatGPT or a small number of general-purpose models in settings such as deep brain stimulation consultation, guideline-based question answering, and relatively small professional test sets (17, 18). Their restricted model coverage and narrow task scope make it difficult to determine whether different LLMs perform consistently across factual accuracy, response quality, and clinical reasoning, or whether their relative strengths vary by knowledge domain, difficulty level, and response structure. More broadly, specialty-specific professional benchmarks have begun to emerge (8, 9, 19, 20), but systematic multidimensional evaluations in disease-specific professional medical settings remain limited. These evidence gaps not only limit understanding of model capabilities but also make it difficult for medical educators and clinicians to select appropriate models for specific tasks and to identify applications requiring more stringent human oversight (12, 15, 16).

To address these gaps, we compared nine LLMs using a PD-specific professional question set covering different clinical domains, difficulty levels, and question types. Responses were evaluated for expert-rated factual accuracy, clinical reasoning, overall quality, and linguistic complexity. We aimed to characterize differences in model performance across these dimensions and examine their relationships.

## Methods

### Study design and question set

This cross-sectional comparative study evaluated nine large language models (LLMs) using 200 English-language professional questions on PD, yielding 1,800 model-generated responses. To minimize the risk of benchmark contamination, publicly available medical question banks and commonly used LLM benchmark datasets were not used. Instead, a *de novo* PD-specific benchmark was developed based on current PD clinical guidelines, expert consensus statements, specialist textbooks, and professional educational materials. All questions and reference answers were independently reviewed by three neurologists with expertise in movement disorders to ensure clinical accuracy and relevance. The questions covered five prespecified domains: pathogenesis, diagnosis and differential diagnosis, treatment, clinical reasoning, and safety and ethics, with 40 questions in each domain. Difficulty was classified as elementary (*n* = 38), intermediate (*n* = 50), advanced (*n* = 51), or expert (*n* = 61). Difficulty was determined by the level of domain knowledge and clinical reasoning required for a correct response. Elementary items involved direct recall of fundamental or widely established PD knowledge. Intermediate items required application of established knowledge to routine clinical questions. Advanced items required integration of several relevant clinical considerations or discrimination between competing diagnostic or management options. Expert items involved specialized knowledge, uncommon clinical scenarios, or complex decision-making for which subspecialty expertise would typically be expected. The benchmark included 157 closed-ended questions, comprising 49 true/false, 81 single-choice, and 27 multiple-choice questions, as well as 43 open-ended questions. Among the 81 single-choice questions, seven were based on clinical vignettes and were additionally classified as case-based single-choice questions and analyzed as an exploratory subset.

This study did not involve human participants or identifiable patient data. All analyses were based exclusively on model-generated outputs; therefore, ethics committee or institutional review board approval was not required.

### Model interaction and data collection

Nine LLMs were evaluated, including GPT-5.5, Kimi-K2.6, Qwen3.6-plus, GLM-5.1, Qwen3.6-flash, DeepSeek-v4-pro, Doubao-Seed-2.0-pro, DeepSeek-v4-flash, and Doubao-Seed-2.0-lite. Model evaluations were conducted between July 20 and July 27, 2026. Model-specific API providers, API model identifiers and endpoints, account/service tiers, reasoning modes, generation settings, browsing/tool settings, and available date/time records are summarized in Supplementary Table S1.

All requests were submitted programmatically through the official provider APIs using a standardized zero-shot protocol. Each model–question pair was issued as an independent API request to prevent conversational carryover. One response was collected for each model–question pair, yielding 1,800 responses in total. Only the initial response was retained; no follow-up prompting, regeneration, or manual revision was permitted. Supplementary Data S2 contains the verbatim initial API outputs without content-level post-processing.

The same standardized prompt template was used for all question types. The complete prompt template was as follows:

“*Assume you are an expert in the field of Parkinson’s disease and answer the following question in English:*

*Return the results strictly in the following format:*

*Correct Answer: (Provide the correct answer directly).*

*Analysis: (Present the thinking process for answering).*

*Question:[question content].”*

The only variable component of the prompt was [question content]. For closed-ended items, this field contained the complete question stem and all response options verbatim; for open-ended items, it contained the complete question stem only. The labels “Correct Answer:” and “Analysis:” were explicitly required by the prompt and were not introduced during post-processing. No explicit word- or sentence-length restriction was imposed. API-level generation settings were recorded where available and are summarized in Supplementary Table S1.

No web-search, browsing, retrieval-augmented generation, external retrieval, or auxiliary tool calls were attached to the API requests. Model identity was determined from the exact API model identifier specified in each request and, where available, corroborated by returned API metadata. Provider-side backend snapshots, routing behavior, and updates that were not exposed through the API could not be independently verified. No refusals, truncated responses, or technical generation failures occurred during the primary evaluation.

To assess stochastic response stability, a repeated-generation robustness analysis was additionally performed on a purposive subset of 10 benchmark items. Each item was independently submitted three times to all nine model configurations, yielding 270 repeated outputs. All repeated generations were conducted as independent, stateless API requests using the identical prompt template and the same model-specific generation settings as in the primary evaluation. The three runs for each model–question pair were conducted approximately 2–5 min apart within the same evaluation session, with no conversational context or prior output carried across runs. No web search, retrieval, or auxiliary tools were enabled during repeated generation. Repeated responses were classified as Consistent, Minor variation, or Inconsistent according to whether differences across runs altered the clinical conclusion. Full procedures and results are provided in Supplementary Data S3.

### Response evaluation

Before evaluation, all model responses were de-identified with respect to model identity and presented to the raters in a randomized order. The raters were blinded to which LLM generated each response throughout the scoring process. All model responses were independently evaluated by two movement-disorder specialists using three prespecified dimensions: expert-rated factual accuracy, clinical reasoning, and Global Quality Scale (GQS), each rated on a 5-point Likert scale. Detailed scoring criteria are provided in Supplementary Table S2.

Expert-rated factual accuracy assessed the factual correctness of the complete response, taking into account both the final answer and its accompanying explanation rather than answer-option correctness alone. Clinical Reasoning assessed the coherence and appropriateness of the reasoning process and the integration of clinically relevant information, whereas GQS assessed the overall quality, completeness, clarity, and usefulness of the response. Higher scores indicated better performance. Readability was assessed using the Flesch–Kincaid Grade Level (FKGL), calculated using Python version 3.11.4; lower FKGL values indicate easier readability. Inter-rater reliability for manually rated outcomes was assessed using quadratically weighted Cohen’s κ, exact agreement, and agreement within one scale point. Disagreements between the two primary raters were independently adjudicated by a third movement-disorder specialist. Following adjudication, a single final rating was assigned for each manually rated outcome and used in the primary analyses.

As a complementary *post hoc* analysis, final-answer correctness was evaluated for the 157 closed-ended items using normalized exact-match scoring. For True/False items, explicit binary judgments or unambiguous narrative affirmations/negations were normalized to True or False. Single-choice and case-based single-choice items required an exact option match, while multiple-choice items required an exact match of the complete option set; partial matches were scored as incorrect. This analysis was separate from the 5-point expert-rated factual accuracy score.

### Statistical analysis

Because all nine models answered the same 200 questions, Question ID was treated as the blocking unit. Between-model differences in expert-rated factual accuracy, clinical reasoning, GQS, and FKGL were assessed using Friedman tests with Kendall’s W; 95% CIs were estimated by 5,000 question-level bootstrap resamples. Stratified analyses were conducted by response structure, question format, PD domain, and difficulty level, with Benjamini–Hochberg adjustment applied separately to domain and difficulty analyses. Three prespecified within-family contrasts were tested using paired Wilcoxon signed-rank tests with Holm correction within each outcome. Supplementary analyses included Spearman correlations with question-cluster bootstrap 95% CIs and response-length sensitivity models using log-transformed word count, model and Question ID fixed effects, and Question ID–clustered robust standard errors. All tests were two-sided with *p* < 0.05 considered significant. Analyses were performed using Python 3.11.4. Score distributions for the three expert-rated outcomes were summarized as counts and percentages at each 1–5 score level. Between-model differences in the complementary paired binary final-answer correctness outcome were evaluated using Cochran’s Q test.

## Results

### Dataset characteristics and rating reliability

All nine models completed the 200-item Parkinson’s disease (PD) question set, yielding 1,800 analyzable responses. The benchmark comprised 157 closed-ended and 43 open-ended questions, spanned five PD knowledge domains, and included 38 elementary, 50 intermediate, 51 advanced, and 61 expert-level items. Inter-rater agreement based on the two independent pre-adjudication ratings was high across all manually rated outcomes. Quadratically weighted Cohen’s κ was 0.876 for GQS (89.9% exact agreement), 0.861 for Clinical Reasoning (87.7% exact agreement), and 0.780 for Expert-rated factual accuracy (88.4% exact agreement). All paired ratings for Expert-rated factual accuracy, Clinical Reasoning, and GQS differed by no more than one scale point. FKGL was calculated algorithmically and therefore did not require inter-rater reliability assessment.

A repeated-generation robustness analysis was conducted on the selected 10-item subset. All nine model configurations were evaluated across 10 questions with three independent runs per item, yielding 270 repeated outputs and 90 question–model pairs. Of these pairs, 69 (76.7%) were classified as Consistent, 18 (20.0%) showed Minor variation without a change in the clinical conclusion, and 3 (3.3%) were Inconsistent, corresponding to an overall clinical-conclusion stability of 96.7%. Full repeated outputs and model-level stability results are provided in Supplementary Data S3.

### Between-model separation varied by evaluation dimension and response structure

Question-paired Friedman analyses identified significant between-model differences across all four prespecified outcomes, but the magnitude of separation varied substantially. As shown in Figure 1A, expert-rated factual accuracy showed limited between-model separation (Kendall’s W = 0.031, bootstrap 95% CI 0.019–0.056), whereas FKGL (W = 0.235, 95% CI 0.206–0.272) and clinical reasoning (W = 0.256, 95% CI 0.223–0.297) showed greater separation. GQS yielded the greatest between-model separation (W = 0.301, 95% CI 0.264–0.347).

**Figure 1:**
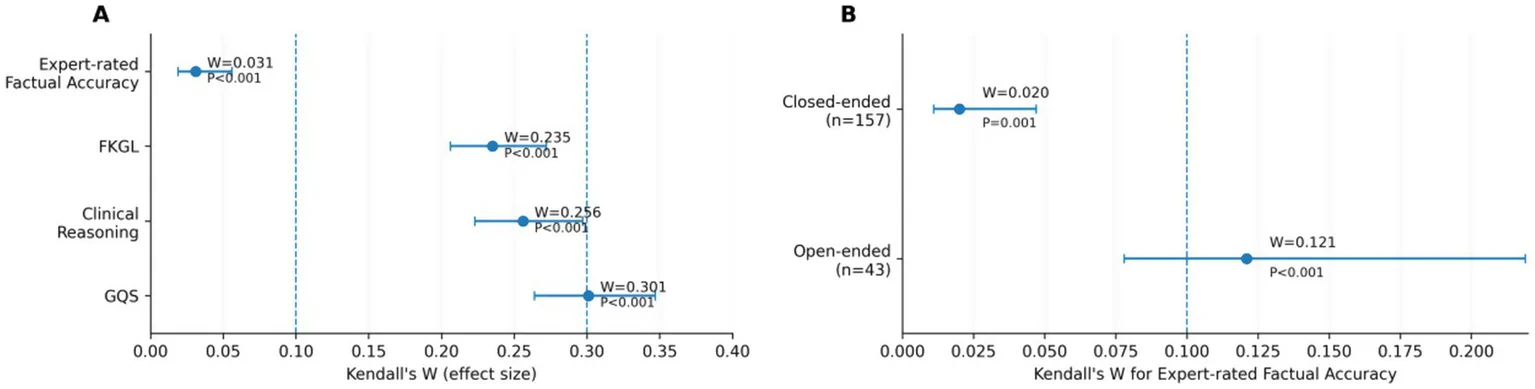
Between-model separation across evaluation dimensions and response structure. **(A)** Kendall’s W for expert-rated factual accuracy, FKGL, Clinical Reasoning, and GQS; points show effect-size estimates and horizontal bars show question-level bootstrap 95% CIs. Displayed *p* values are from Friedman tests. Dashed reference lines at W = 0.10 and W = 0.30 are descriptive only. **(B)** Between-model separation in expert-rated factual accuracy heterogeneity for closed-ended and open-ended questions, with question-level bootstrap 95% CIs; the dashed line at W = 0.10 is a descriptive reference point.

As shown in Figure 1B and Table 1, between-model separation in expert-rated factual accuracy was greater for open-ended than for closed-ended questions. Across the 157 closed-ended questions, between-model separation was statistically detectable but small in magnitude (Friedman χ^2^ = 25.50, df = 8, *p* = 0.001, W = 0.020; bootstrap 95% CI 0.011–0.047). In contrast, the 43 open-ended questions showed greater separation (χ^2^ = 41.63, df = 8, *p* < 0.001, W = 0.121; 95% CI 0.078–0.219). In format-specific analyses, between-model separation in expert-rated factual accuracy remained small for true/false (W = 0.036, *p* = 0.080), single-choice (W = 0.031, *p* = 0.009), and multiple-choice questions (W = 0.021, *p* = 0.812), whereas greater separation was observed for open-ended questions (W = 0.121, *p* < 0.001). The seven case-based single-choice questions were examined separately as an exploratory contextual subset (W = 0.181, *p* = 0.256).

**Table 1:** Between-model separation in expert-rated factual accuracy by response structure and question format.

Response structure / format	Questions (*n*)	Friedman χ^2^	*p*	Kendall’s W
All questions	200	50.14	<0.001***	0.031
Closed-ended	157	25.50	0.001**	0.020
True/false	49	14.05	0.080	0.036
Single-choice (incl. Case-based)	81	20.26	0.009**	0.031
Case-based single-choice subset	7	10.13	0.256	0.181
Multiple-choice	27	4.48	0.812	0.021
Open-ended	43	41.63	<0.001***	0.121

All tests used Question ID as the blocking unit. The case-based single-choice analysis is exploratory because *n* = 7 and overlaps with the single-choice category. * *p* < 0.05, ** *p* < 0.01, ***p < 0.001.

Score distributions are summarized in Supplementary Figure S4. For expert-rated factual accuracy, score 4 accounted for 73.5–88.0% of each model’s responses, indicating substantial concentration within a narrow part of the 5-point scale. In the complementary final-answer correctness analysis of the 157 closed-ended items (1,413 model responses), 1,373 responses (97.2%) met the normalized exact-match criterion. Model-specific correctness ranged from 96.2 to 98.7%, with no significant overall between-model difference (Cochran’s Q = 6.369, df = 8, *p* = 0.606; Supplementary Figure S5). Full item-level coding and scoring rules are provided in Supplementary Data S5.

### Comparable factual performance masked distinct multidimensional response profiles

As summarized in Table 2 and visualized in Figure 2, substantial differences were evident in other dimensions of response performance. Gpt-5.5 had the highest mean Expert-rated factual accuracy (3.96, 95% CI 3.89–4.03) and GQS (4.71, 95% CI 4.62–4.79), while also showing the lowest FKGL (18.46, 95% CI 18.09–18.83). Qwen3.6-plus achieved the highest Clinical Reasoning score (4.76, 95% CI 4.68–4.83), and Kimi-K2.6 also performed highly on Clinical Reasoning (4.69, 95% CI 4.61–4.77) and GQS (4.65, 95% CI 4.58–4.72).

**Table 2:** Model performance on the original measurement scales.

Model	Expert-rated factual accuracy	Clinical reasoning	GQS	FKGL
Gpt-5.5	3.96 (3.89–4.03)	4.75 (4.67–4.82)	4.71 (4.62–4.79)	18.46 (18.09–18.83)
Qwen3.6-plus	3.88 (3.81–3.93)	4.76 (4.68–4.83)	4.67 (4.60–4.75)	23.70 (23.11–24.33)
Kimi-K2.6	3.89 (3.83–3.95)	4.69 (4.61–4.77)	4.65 (4.58–4.72)	20.49 (20.04–20.95)
Qwen3.6-flash	3.71 (3.62–3.80)	4.40 (4.28–4.52)	4.44 (4.34–4.54)	24.32 (23.72–25.06)
GLM-5.1	3.85 (3.78–3.92)	4.45 (4.36–4.54)	4.39 (4.31–4.48)	19.98 (19.57–20.38)
Deepseek-v4-pro	3.84 (3.77–3.91)	4.35 (4.25–4.45)	4.28 (4.19–4.36)	21.13 (20.63–21.62)
Doubao-Seed-2.0-pro	3.89 (3.83–3.94)	4.36 (4.28–4.44)	4.26 (4.18–4.33)	22.48 (21.64–23.31)
Doubao-Seed-2.0-lite	3.83 (3.76–3.91)	4.09 (4.01–4.16)	4.01 (3.94–4.08)	21.87 (21.09–22.69)
Deepseek-v4-flash	3.80 (3.71–3.88)	4.03 (3.95–4.11)	3.94 (3.86–4.01)	20.63 (20.07–21.21)

Values are means with question-level bootstrap 95% confidence intervals. Lower FKGL indicates easier readability.

**Figure 2:**
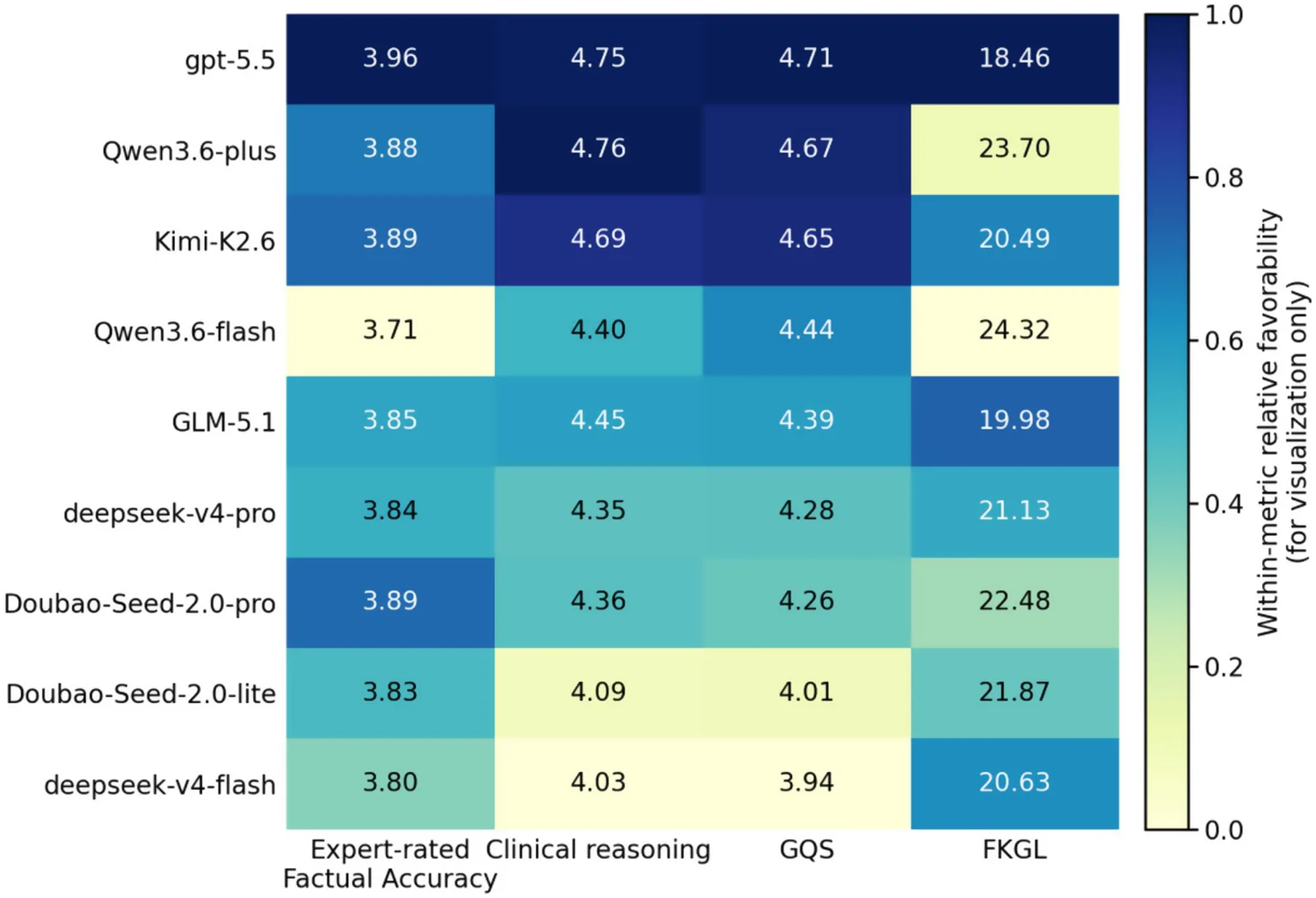
Capability profiles across the nine models. Cell values are raw means on the original measurement scales. Cell shading represents within-metric relative favorability for visualization only; FKGL is direction-reversed because lower values indicate easier readability. No outcomes were combined or weighted.

Figure 2 further illustrates that similar expert-rated factual accuracy did not necessarily correspond to similar clinical response quality. Kimi-K2.6 and Doubao-Seed-2.0-pro had the same mean expert-rated factual accuracy (3.89), but their descriptive multidimensional profiles differed: Kimi-K2.6 showed higher mean Clinical Reasoning (4.69 vs. 4.36) and GQS (4.65 vs. 4.26). These descriptive contrasts illustrate that similar Expert-rated factual accuracy can coexist with different response-quality profiles.

Supplementary analyses further examined relationships among evaluation dimensions and response length. Clinical Reasoning and GQS were strongly correlated (Spearman rho = 0.747, question-cluster bootstrap 95% CI 0.711–0.782), whereas Expert-rated factual accuracy showed weaker associations with Clinical Reasoning (rho = 0.335) and GQS (rho = 0.324), and associations involving FKGL were weak (Supplementary Figure S1; Supplementary Table S3). In response-length sensitivity models, longer responses were associated with higher Clinical Reasoning (standardized beta = 0.554, 95% CI 0.441–0.668, *p* < 0.001) and GQS (beta = 0.683, 95% CI 0.560–0.806, *p* < 0.001), but not with Expert-rated factual accuracy or FKGL (Supplementary Figure S2; Supplementary Table S6).

### Between-model separation varied across PD domains and difficulty

Context-specific analyses preserved Question ID as the blocking unit. As shown in Figure 3A and Supplementary Table S4, expert-rated factual accuracy effects remained negligible (W = 0.017–0.074). By contrast, clinical reasoning showed W = 0.230–0.318, GQS ranged from 0.175 to 0.431, and FKGL from 0.220 to 0.293. The largest domain-specific separation was observed for GQS in Safety & Ethics (W = 0.431), followed by GQS in Treatment (W = 0.378) and in the Clinical Reasoning domain (W = 0.351).

**Figure 3:**
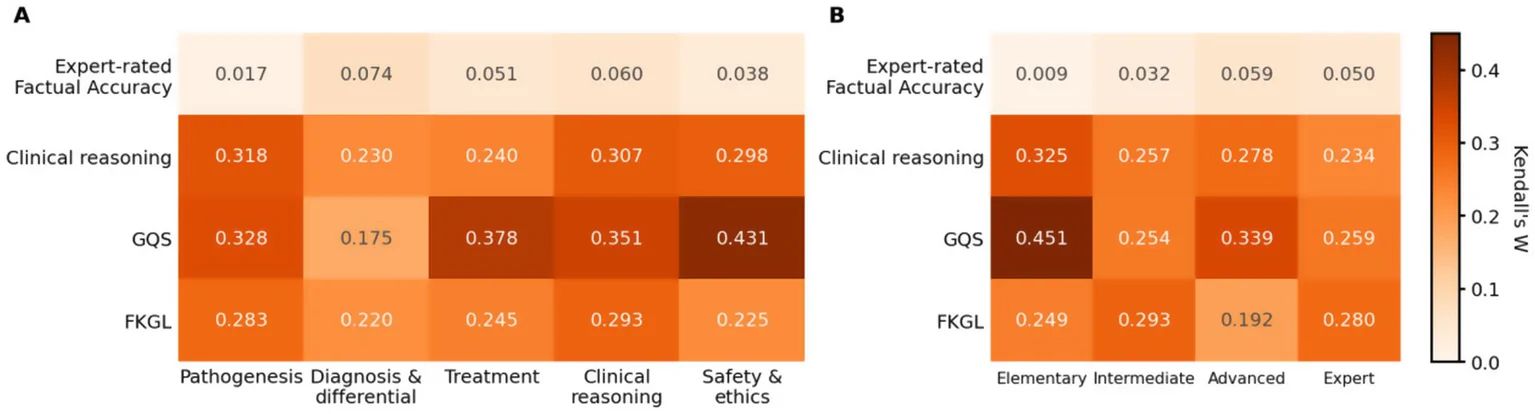
Context-specific between-model separation. **(A)** Kendall’s W across the five PD knowledge domains and **(B)** across the four difficulty levels. A common color scale is used across all outcomes; higher values indicate greater between-model separation. Corresponding FDR-adjusted *p* values and bootstrap 95% CIs are reported in Supplementary Table S4.

Because the Safety & Ethics domain showed the largest GQS between-model separation, model-specific performance was examined descriptively. Qwen3.6-plus and gpt-5.5 had the highest mean GQS (4.80 and 4.72, respectively) and Clinical Reasoning scores (4.78 and 4.72), whereas mean Expert-rated factual accuracy remained tightly clustered across models (3.85–4.03) (Supplementary Figure S3; Supplementary Table S5).

As shown in Figure 3B and Supplementary Table S4, a similar pattern was observed across difficulty levels. Expert-rated factual accuracy remained weakly discriminative at all four levels (W = 0.009–0.059), including advanced and expert questions. In contrast, GQS showed substantially larger effects (W = 0.254–0.451). Notably, the greatest GQS separation occurred for elementary rather than advanced or expert questions (W = 0.451). Clinical reasoning ranged from W = 0.234 to 0.325 and FKGL from W = 0.192 to 0.293. Between-model separation in expert-rated factual accuracy did not show a consistent increase with question difficulty.

### Flagship variants showed dimension-specific performance advantages

Within-family comparisons were treated as version contrasts rather than causal interventions. As shown in Figure 4 and Table 3, relative to their corresponding standard or lightweight variants, all three flagship variants had higher Clinical Reasoning and GQS scores after Holm correction (Figures 4B,C). DeepSeek-v4-pro and Doubao-Seed-2.0-pro did not show significant Expert-rated factual accuracy differences from their comparators, whereas Qwen3.6-plus had higher Expert-rated factual accuracy than Qwen3.6-flash (mean paired difference +0.165, Holm-adjusted *p* < 0.001) (Figure 4A). For FKGL, all three within-family contrasts were statistically significant after Holm correction, but the directions differed (Figure 4D). DeepSeek-v4-pro had a higher FKGL than deepseek-v4-flash (21.13 vs. 20.63, Holm-adjusted *p* = 0.045), and Doubao-Seed-2.0-pro had a higher FKGL than Doubao-Seed-2.0-lite (22.48 vs. 21.87, Holm-adjusted *p* = 0.045), whereas Qwen3.6-plus had a lower FKGL than Qwen3.6-flash (23.70 vs. 24.32, Holm-adjusted *p* = 0.045). The largest version contrasts on the 5-point expert-rated dimensions were Qwen Clinical Reasoning (+0.355) and DeepSeek GQS (+0.340).

**Figure 4:**
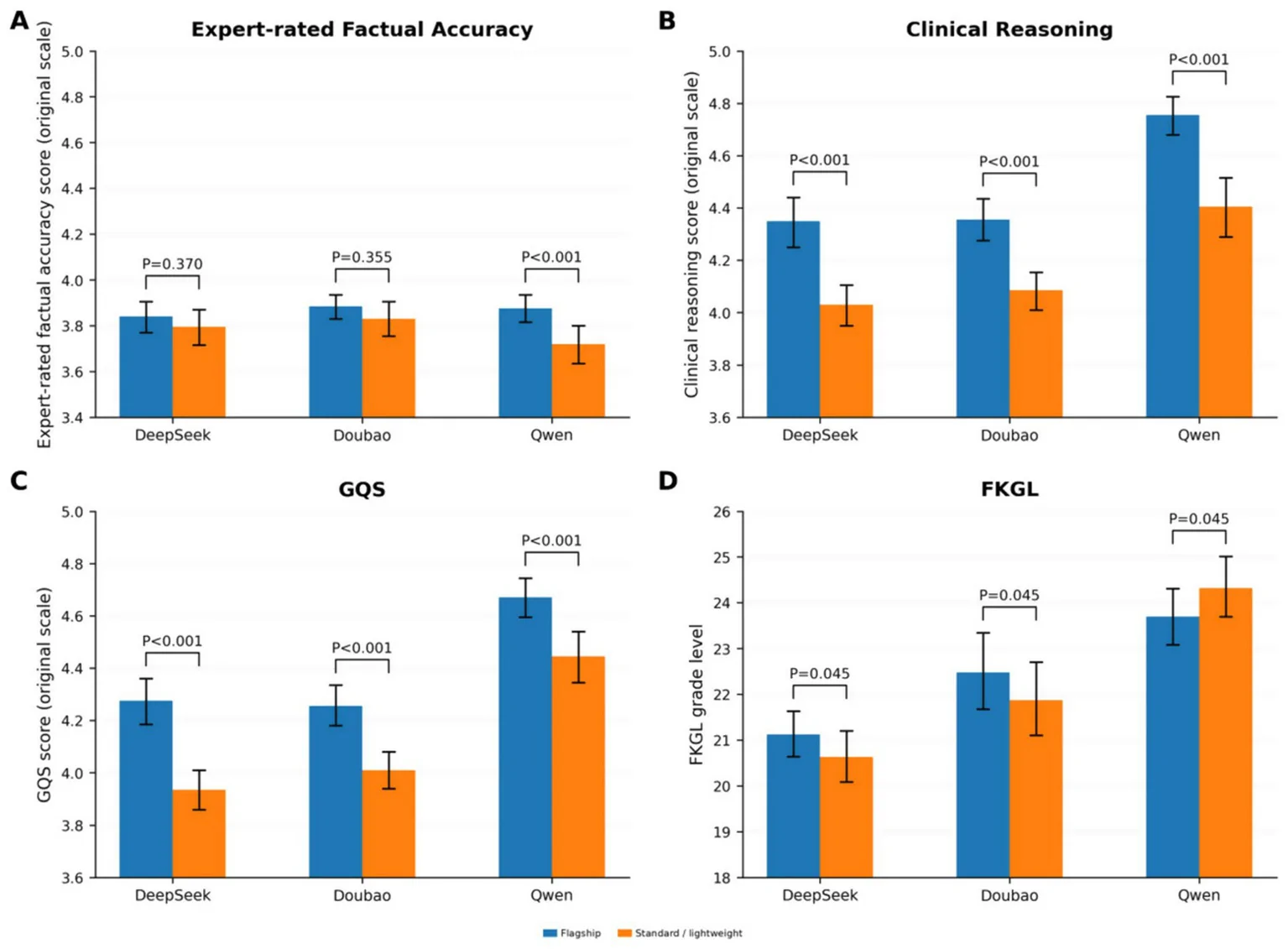
Within-family flagship versus standard/lightweight model comparisons for **(A)** Expert-rated factual accuracy, **(B)** Clinical Reasoning, **(C)** GQS, and **(D)** FKGL. Bars show model means and error bars show question-level bootstrap 95% CIs. Displayed *p* values are Holm-adjusted values from paired Wilcoxon signed-rank tests. Lower FKGL indicates easier readability.

**Table 3:** Paired flagship-versus-comparator contrasts.

Family	Outcome	Mean difference	95% CI	Rank-biserial	Holm P
Deepseek	Expert-rated factual accuracy	0.045	−0.040–0.135	0.143	0.370
	Clinical reasoning	0.320	0.220–0.425	0.613	<0.001***
	GQS	0.340	0.245–0.430	0.751	<0.001***
	FKGL	0.496	−0.153–1.134	0.198	0.045*
Doubao	Expert-rated factual accuracy	0.055	−0.020–0.130	0.211	0.355
	Clinical reasoning	0.270	0.180–0.360	0.653	<0.001***
	GQS	0.245	0.175–0.320	0.858	<0.001***
	FKGL	0.608	−0.265–1.492	0.196	0.045*
Qwen	Expert-rated factual accuracy	0.165	0.085–0.250	0.516	<0.001***
	Clinical reasoning	0.355	0.240–0.475	0.724	<0.001***
	GQS	0.230	0.140–0.325	0.673	<0.001***
	FKGL	−0.628	−1.312–0.015	−0.173	0.045*

* *p* < 0.05, ** *p* < 0.01, ***p < 0.001. Confidence intervals are question-level bootstrap intervals for the mean paired difference, whereas *p* values are from paired Wilcoxon signed-rank tests with Holm adjustment; therefore, CI inclusion of zero and statistical significance need not correspond exactly.

All differences on the 5-point expert-rated dimensions were below 0.4 points. Because no validated clinically perceptible threshold was prespecified, statistical significance was not interpreted as evidence of clinical importance.

## Discussion

This study compared nine contemporary large language models (LLMs) across 200 professional Parkinson’s disease (PD) questions using a multidimensional evaluation framework. Between-model separation in overall expert-rated factual accuracy was limited (Kendall’s W = 0.031), whereas greater separation was observed for Clinical Reasoning, GQS, and FKGL. Expert-rated factual accuracy showed particularly limited separation on closed-ended questions (W = 0.020), while open-ended questions produced greater separation (W = 0.121). This pattern was accompanied by a concentration of factual-accuracy ratings at score 4 and high closed-ended final-answer correctness across models (96.2–98.7%), suggesting that the small W may partly reflect restricted discrimination within this benchmark and scoring framework rather than model equivalence. These findings indicate that factual accuracy alone may provide an incomplete account of model performance, and that reasoning, overall response quality, and readability offer additional discriminatory value.

This pattern is consistent with the recent shift in medical LLM evaluation beyond examination-style accuracy toward more clinically oriented, multidimensional assessment (21–23). Closed-ended questions are useful for testing whether a model can retrieve and provide correct information, but they are less suited to assessing information integration, explanation, uncertainty management, and appropriate qualification. In our study, although expert-rated factual accuracy differences among models were statistically detectable for closed-ended questions, the effect size was negligible. Open-ended questions showed greater model separation. The seven case-based single-choice questions also produced a larger point estimate (W = 0.181), but the omnibus comparison was not significant (*p* = 0.256), and the small size of this exploratory subset precludes firm conclusions. In this benchmark, closed-ended items produced less between-model separation than open-ended items. Whether expanding the number, difficulty, or design of closed-ended questions would improve discrimination requires further investigation. Tasks requiring explanation, comparison of alternatives, and contextual judgment may therefore provide greater evaluative value.

PD is particularly suitable for examining this distinction because diagnosis and treatment rarely depend on a single fact. Clinical decisions often require integration of disease course, symptom characteristics, levodopa responsiveness, cognitive and psychiatric status, treatment-related adverse effects, and individual patient goals (1, 2, 4). A treatment that improves motor symptoms, for example, may require a very different risk–benefit assessment in a patient with hallucinations, orthostatic hypotension, cognitive impairment, or impulse-control disorders. Thus, factual difficulty is not necessarily equivalent to clinical complexity. This interpretation is consistent with our stratified analyses. Expert-rated factual accuracy remained weakly discriminative across PD domains and difficulty levels, whereas GQS showed much greater separation in Safety & Ethics (W = 0.431), Treatment (W = 0.378), and Clinical Reasoning (W = 0.351). Notably, the largest GQS separation occurred among elementary questions (W = 0.451), rather than advanced or expert questions. A rare but well-defined fact may be difficult mainly because the knowledge is obscure, whereas an apparently elementary question involving hallucinations, cognitive decline, medication adverse effects, contraindications, or treatment trade-offs may require considerably more contextual judgment. Previous PD-specific LLM studies have likewise reported that otherwise favorable responses may still be limited by incomplete information, poor readability, or overly definitive recommendations (17, 18). Evaluation in PD should therefore consider not only whether the information is correct, but also whether clinically relevant risks, alternatives, and uncertainty are communicated appropriately.

Another important finding was that the relative performance of models varied across evaluation dimensions. Models with similar expert-rated factual accuracy could differ noticeably in clinical reasoning and GQS. Similarly, flagship variants in all three model families showed higher Clinical Reasoning and GQS than their corresponding standard or lightweight variants, whereas a significant expert-rated factual accuracy advantage was observed only within the Qwen family. These differences should not, however, be interpreted as evidence of clinical superiority. All flagship-versus-comparator differences on the 5-point expert-rated outcomes were below 0.4 points, and no threshold for a clinically meaningful difference was prespecified. This illustrates why a single accuracy-based leaderboard may be insufficient for characterizing models intended for different medical tasks.

From a biomedical informatics perspective, model-level benchmark performance should also not be equated with system-level clinical utility. Friedman’s fundamental framework emphasizes that the value of an information resource depends not only on the resource itself, but also on whether a person working with it performs better than the person alone (24). A model with high factual accuracy may therefore still provide limited practical value if its information organization, risk qualification, readability, or fit with the clinical workflow is poor. This is particularly relevant to medical information systems, in which knowledge retrieval, patient education, treatment explanation, and complex decision support impose different requirements for accuracy, reasoning, readability, and safety. Randomized and systematic evidence similarly indicates that strong standalone LLM performance does not necessarily translate into better user decisions or workflow outcomes, and that the benefit of human–LLM collaboration depends on task design and implementation context (25–27). Accordingly, our findings are better viewed as pre-implementation characterization of model performance than as evidence for direct deployment. Future system-level studies should test whether different PD tasks require different model profiles and whether high-risk queries benefit from guideline retrieval, structured safeguards, or human review.

Readability further illustrates this multidimensional trade-off. FKGL varied appreciably across models, and flagship variants did not consistently show better readability. This matters because the same medical information system may be used by movement-disorder specialists, general neurologists, trainees, patients, and caregivers. A detailed explanation appropriate for a specialist may be unnecessarily difficult for a patient, whereas excessive simplification may omit important risks. Clinical Reasoning and GQS were strongly correlated in our study (ρ = 0.747), whereas expert-rated factual accuracy showed weaker associations with both measures. Response length was also positively associated with Clinical Reasoning and GQS but not with expert-rated factual accuracy. Longer responses should therefore not be assumed to be more accurate or more clinically useful. Future evaluations may benefit from considering not only completeness, but also how efficiently task-relevant and clinically useful information is communicated.

## Limitations

Several limitations should be acknowledged. First, the benchmark did not fully reflect real-world clinical decision-making, particularly given the limited number of case-based questions. Second, Clinical Reasoning and GQS involved subjective expert ratings, while FKGL measures textual complexity rather than actual usability. Third, the primary analysis retained one response per model–question pair. Although a repeated-generation analysis on a 10-item subset provided a limited robustness assessment, it does not fully characterize stochastic variability across the entire 200-item benchmark. Finally, this benchmark did not evaluate workflow integration, output-format compliance, token/cost efficiency, response latency, clinician decision-making, or patient outcomes. Future prospective API-based evaluations should incorporate these system-level measures where feasible.

## Conclusion

Contemporary LLMs showed limited between-model separation in expert-rated factual accuracy within this benchmark and scoring framework on professional PD questions, but more substantial differences remained in Clinical Reasoning, GQS, and readability, and open-ended tasks provided greater discrimination than closed-ended tasks. For medical information systems, multidimensional benchmarking may be most useful for characterizing model performance across specific task requirements and informing subsequent evaluation of human–AI collaboration, workflow integration, and system-level validation.

## Data Availability

The original contributions presented in the study are included in the article/Supplementary material, further inquiries can be directed to the corresponding author.

## References

[1] Bloem BR Okun MS Klein C. Parkinson's disease. Lancet. (2021) 397:2284–303. doi: 10.1016/S0140-6736(21)00218-X, 33848468

[2] Tolosa E Garrido A Scholz SW Poewe W. Challenges in the diagnosis of Parkinson's disease. Lancet Neurol. (2021) 20:385–97. doi: 10.1016/S1474-4422(21)00030-2, 33894193 PMC8185633

[3] Armstrong MJ Okun MS. Diagnosis and treatment of Parkinson disease: a review. JAMA. (2020) 323:548–60. doi: 10.1001/jama.2019.22360, 32044947

[4] Foltynie T Bruno V Fox S Kühn AA Lindop F Lees AJ. Medical, surgical, and physical treatments for Parkinson's disease. Lancet. (2024) 403:305–24. doi: 10.1016/S0140-6736(23)01429-0, 38245250

[5] Moor M Banerjee O Abad ZSH Krumholz HM Leskovec J Topol EJ . Foundation models for generalist medical artificial intelligence. Nature. (2023) 616:259–65. doi: 10.1038/s41586-023-05881-4, 37045921

[6] Singhal K Azizi S Tu T Mahdavi SS Wei J Chung HW . Large language models encode clinical knowledge. Nature. (2023) 620:172–80. doi: 10.1038/s41586-023-06291-2, 37438534 PMC10396962

[7] Kung TH Cheatham M Medenilla A Sillos C De Leon L Elepaño C . Performance of ChatGPT on USMLE: potential for AI-assisted medical education using large language models. PLoS Digit Health. (2023) 2:e0000198. doi: 10.1371/journal.pdig.0000198, 36812645 PMC9931230

[8] Xu P Wu Y Jin K Chen X He M Shi D. DeepSeek-R1 outperforms Gemini 2.0 pro, OpenAI o1, and o3-mini in bilingual complex ophthalmology reasoning. Adv Ophthalmol Pract Res. (2025) 5:189–95. doi: 10.1016/j.aopr.2025.05.001, 40678192 PMC12269606

[9] Arslan İ Terzi Kumandaş M Arslan D Aslan Koca K Saltoğlu T Çalışkan SA . Humans vs. large language models in neurology board examination: performance, limitations, and reference reliability. Acta Neurol Belg. (2026) 126:1401–12. doi: 10.1007/s13760-026-03102-z, 42240737

[10] Thirunavukarasu AJ Ting DSJ Elangovan K Gutierrez L Tan TF Ting DSW. Large language models in medicine. Nat Med. (2023) 29:1930–40. doi: 10.1038/s41591-023-02448-8, 37460753

[11] Lee P Bubeck S Petro J. Benefits, limits, and risks of GPT-4 as an AI Chatbot for medicine. N Engl J Med. (2023) 388:1233–9. doi: 10.1056/NEJMsr2214184, 36988602

[12] Hager P Jungmann F Holland R Bhagat K Hubrecht I Knauer M . Evaluation and mitigation of the limitations of large language models in clinical decision-making. Nat Med. (2024) 30:2613–22. doi: 10.1038/s41591-024-03097-1, 38965432 PMC11405275

[13] Goh E Gallo R Hom J Strong E Weng Y Kerman H . Large language model influence on diagnostic reasoning: a randomized clinical trial. JAMA Netw Open. (2024) 7:e2440969. doi: 10.1001/jamanetworkopen.2024.40969, 39466245 PMC11519755

[14] Singhal K Tu T Gottweis J Sayres R Wulczyn E Amin M . Toward expert-level medical question answering with large language models. Nat Med. (2025) 31:943–50. doi: 10.1038/s41591-024-03423-7, 39779926 PMC11922739

[15] Lucas HC Upperman JS Robinson JR. A systematic review of large language models and their implications in medical education. Med Educ. (2024) 58:1276–85. doi: 10.1111/medu.15402, 38639098

[16] Haltaufderheide J Ranisch R. The ethics of ChatGPT in medicine and healthcare: a systematic review on large language models (LLMs). NPJ Digit Med. (2024) 7:183. doi: 10.1038/s41746-024-01157-x, 38977771 PMC11231310

[17] Shin D Tang T Carson J Isaac R Dinh C Im D . Subthalamic nucleus or globus pallidus internus deep brain stimulation for the treatment of parkinson's disease: an artificial intelligence approach. J Clin Neurosci. (2025) 138:111393. doi: 10.1016/j.jocn.2025.111393, 40554303

[18] Riccio G Coppola R Chiariello MR Giglio A De Rosa A. Quality and readability of ChatGPT performance on Parkinson's disease. Mov Disord Clin Pract. (2026) [Online ahead of print]. doi: 10.1002/mdc3.70700, 42249802 PMC13338990

[19] Aykac K Cubuk O Demir OO Choe YJ Aydin M Ozsurekci Y. Comparing ChatGPT-3.5, Gemini 2.0, and DeepSeek V3 for pediatric pneumonia learning in medical students. Sci Rep. (2025) 15:40342. doi: 10.1038/s41598-025-27722-2, 41254112 PMC12627666

[20] Liu M Okuhara T Dai Z Huang W Gu L Okada H . Evaluating the effectiveness of advanced large language models in medical knowledge: a comparative study using Japanese national medical examination. Int J Med Inform. (2024) 193:105673. doi: 10.1016/j.ijmedinf.2024.105673, 39471700

[21] Bedi S Cui H Fuentes M Unell A Wornow M Banda JM . Holistic evaluation of large language models for medical tasks with MedHELM. Nat Med. (2026) 32:943–51. doi: 10.1038/s41591-025-04151-2, 41559415 PMC13267972

[22] Rao AS Esmail KP Lee RS Jiang S Arraiza Carlo B Gill J . Large language model performance and clinical reasoning tasks. JAMA Netw Open. (2026) 9:e264003. doi: 10.1001/jamanetworkopen.2026.4003, 41973425 PMC13077515

[23] Vishwanath K Alyakin A Ghosh M Hage A Neifert SN Orillac C . General-purpose large language models outperform specialized clinical AI tools on medical benchmarks. Nat Med. (2026) 32:2405–9. doi: 10.1038/s41591-026-04431-5, 42286322 PMC13375586

[24] Friedman CP. A "fundamental theorem" of biomedical informatics. J Am Med Inform Assoc. (2008) 16:169–70. doi: 10.1197/jamia.M3092, 19074294 PMC2649317

[25] Bean AM Payne RE Parsons G Kirk HR Ciro J Mosquera-Gómez R . Reliability of LLMs as medical assistants for the general public: a randomized preregistered study. Nat Med. (2026) 32:609–15. doi: 10.1038/s41591-025-04074-y PMC12920132 41663592

[26] Tao X Zhou S Ding K Li S Li Y Wu B . An LLM chatbot to facilitate primary-to-specialist care transitions: a randomized controlled trial. Nat Med. (2026) 32:934–42. doi: 10.1038/s41591-025-04176-7, 41555035 PMC13004692

[27] Liu Y Carrero ZI Jiang X Ferber D Wölflein G Zhang L . Benchmarking large language model-based agent systems for clinical decision tasks. NPJ Digit Med. (2026) 9:259. doi: 10.1038/s41746-026-02443-6, 41708802 PMC13031301

