# Supplementary material for: Comparative performance of nine large language models on professional Parkinson’s disease tasks: a multidimensional benchmark study
Source: Front Neurol. 2026 Oct 30;17:1970459. doi: 10.3389/fneur.2026.1970459 (PMC13759406; doi:10.3389/fneur.2026.1970459)
Supplement: Supplementary file 1 [file Supplementary_file_1.docx]

**SUPPLEMENTARY RESULTS**

Supplementary analyses were performed to further characterize model performance and the robustness of the main findings. These included analyses of associations among evaluation dimensions, response-length effects, performance across PD domains and difficulty levels, score distributions, and closed-ended final-answer correctness. Additional results on within-model response stability and representative error patterns are also provided.

## Supplementary Figure S1. Associations among evaluation dimensions

As shown in Supplementary Figure S1 and Supplementary Table S3, Clinical Reasoning and GQS were strongly correlated (Spearman rho=0.747, question-cluster bootstrap 95% CI 0.711-0.782), whereas Expert-rated factual accuracy showed only moderate associations with Clinical Reasoning (rho=0.335) and GQS (rho=0.324). Associations involving FKGL were weak.

**Supplementary Figure S1.** Associations among evaluation dimensions. Cells show Spearman correlation coefficients (rho) for Expert-rated factual accuracy, Clinical Reasoning, GQS, and FKGL. Confidence intervals and P values are reported in Supplementary Table S3.

## Supplementary Figure S2. Response-length sensitivity analysis

As shown in Supplementary Figure S2 and Supplementary Table S6, in fixed-effects sensitivity models accounting for model identity and Question ID, longer responses were associated with higher Clinical Reasoning (standardized beta=0.554, 95% CI 0.441-0.668, P<0.001) and GQS (beta=0.683, 95% CI 0.560-0.806, P<0.001), but not with Expert-rated factual accuracy or FKGL. These analyses were considered sensitivity analyses and were not used to modify the primary estimates.

**Supplementary Figure S2.** Response-length sensitivity analysis. Points show standardized coefficients for log-transformed response length from fixed-effects models including model identity and Question ID; horizontal bars show 95% CIs and the vertical line indicates beta=0. Full estimates are reported in Supplementary Table S6.

## Supplementary Figure S3. Safety & Ethics model-specific performance

As summarized in Supplementary Figure S3 and Supplementary Table S5, because Safety & Ethics produced the largest domain-specific GQS heterogeneity (W=0.431), model-specific scores were examined descriptively. Qwen3.6-plus and gpt-5.5 had the highest mean GQS and Clinical Reasoning scores, while Expert-rated factual accuracy remained tightly clustered across models.

**Supplementary Figure S3.** Model-specific Clinical Reasoning and GQS performance in the Safety & Ethics domain. Symbols represent model-level mean scores; connecting lines are included only to facilitate within-model comparison. Numerical results are reported in Supplementary Table S5

**Supplementary Figure S4. Score distributions**

The distributions of the three expert-rated outcomes are shown in Supplementary Figure S4. Expert-rated factual accuracy was strongly concentrated at score 4 across models, with 73.5%-88.0% of responses per model receiving a score of 4. The corresponding distributions for Clinical Reasoning and GQS showed greater use of the upper end of the 5-point scales and clearer between-model differences.

**Supplementary Figure S4.** Distribution of expert-rated scores across models. (A) Expert-rated factual accuracy, (B) Clinical Reasoning, and (C) GQS. Bars show the percentage of each model's 200 responses assigned to scores 1-5. Percentage labels are displayed for larger segments for readability

.

**Supplementary Figure S5. Closed-ended final-answer correctness**

As a complementary post hoc analysis, normalized exact-match final-answer correctness was evaluated for the 157 closed-ended items (1,413 model responses). Overall, 1,373 responses (97.2%) met the normalized exact-match criterion. Model-specific correctness ranged from 96.2% to 98.7%, and the paired omnibus comparison was not significant (Cochran's Q=6.369, df=8, P=0.606). By format, exact-match correctness was 98.2% for True/False, 99.4% for Single-choice, 88.5% for Multiple-choice, and 100.0% for Case analysis items.

**Supplementary Figure S5.** Conventional final-answer correctness for closed-ended questions. (A) Model-level normalized exact-match correctness across the 157 closed-ended items. (B) Correctness by question format. For Multiple-choice items, a response was counted as correct only when the complete selected option set exactly matched the prespecified reference answer; partially correct or incomplete option sets were counted as incorrect. For True/False items, responses were normalized to True or False when the final-answer field contained either an explicit binary judgment or an unambiguous narrative affirmation or negation of the proposition; ambiguous responses were counted as incorrect. Item-level coding and scoring rules are provided in Supplementary Data S5.

**Supplementary Table S3. Associations among evaluation dimensions**

| **Outcome A** | **Outcome B** | **Spearman rho** | **95% CI** | **P** |
| --- | --- | --- | --- | --- |
| Expert-rated factual accuracy | Clinical Reasoning | 0.335 | 0.268-0.399 | <0.001 |
| Expert-rated factual accuracy | GQS | 0.324 | 0.255-0.387 | <0.001 |
| Expert-rated factual accuracy | FKGL | -0.041 | -0.100-0.019 | 0.080 |
| Clinical Reasoning | GQS | 0.747 | 0.711-0.782 | <0.001 |
| Clinical Reasoning | FKGL | 0.067 | 0.013-0.119 | 0.005 |
| GQS | FKGL | 0.095 | 0.038-0.151 | <0.001 |

## Supplementary Table S4. Paired omnibus analyses by PD domain and difficulty

| **Stratum** | **Level** | **Outcome** | **n** | **Kendall W** | **95% CI** | **FDR P** |
| --- | --- | --- | --- | --- | --- | --- |
| Domain | Pathogenesis | Expert-rated factual accuracy | 40 | 0.017 | 0.013 to 0.083 | 0.712 |
| Domain | Pathogenesis | Clinical Reasoning | 40 | 0.318 | 0.249 to 0.423 | <0.001 |
| Domain | Pathogenesis | GQS | 40 | 0.328 | 0.245 to 0.447 | <0.001 |
| Domain | Pathogenesis | FKGL | 40 | 0.283 | 0.226 to 0.389 | <0.001 |
| Domain | Diagnosis & differential diagnosis | Expert-rated factual accuracy | 40 | 0.074 | 0.041 to 0.170 | 0.003 |
| Domain | Diagnosis & differential diagnosis | Clinical Reasoning | 40 | 0.230 | 0.181 to 0.322 | <0.001 |
| Domain | Diagnosis & differential diagnosis | GQS | 40 | 0.175 | 0.123 to 0.273 | <0.001 |
| Domain | Diagnosis & differential diagnosis | FKGL | 40 | 0.220 | 0.161 to 0.323 | <0.001 |
| Domain | Treatment | Expert-rated factual accuracy | 40 | 0.051 | 0.029 to 0.136 | 0.044 |
| Domain | Treatment | Clinical Reasoning | 40 | 0.240 | 0.182 to 0.348 | <0.001 |
| Domain | Treatment | GQS | 40 | 0.378 | 0.311 to 0.466 | <0.001 |
| Domain | Treatment | FKGL | 40 | 0.245 | 0.198 to 0.335 | <0.001 |
| Domain | Clinical reasoning | Expert-rated factual accuracy | 40 | 0.060 | 0.032 to 0.151 | 0.016 |
| Domain | Clinical reasoning | Clinical Reasoning | 40 | 0.307 | 0.240 to 0.412 | <0.001 |
| Domain | Clinical reasoning | GQS | 40 | 0.351 | 0.282 to 0.457 | <0.001 |
| Domain | Clinical reasoning | FKGL | 40 | 0.293 | 0.237 to 0.389 | <0.001 |
| Domain | Safety & ethics | Expert-rated factual accuracy | 40 | 0.038 | 0.020 to 0.123 | 0.152 |
| Domain | Safety & ethics | Clinical Reasoning | 40 | 0.298 | 0.234 to 0.405 | <0.001 |
| Domain | Safety & ethics | GQS | 40 | 0.431 | 0.333 to 0.556 | <0.001 |
| Domain | Safety & ethics | FKGL | 40 | 0.225 | 0.175 to 0.317 | <0.001 |
| Difficulty | Elementary | Expert-rated factual accuracy | 38 | 0.009 | 0.010 to 0.075 | 0.945 |
| Difficulty | Elementary | Clinical Reasoning | 38 | 0.325 | 0.240 to 0.439 | <0.001 |
| Difficulty | Elementary | GQS | 38 | 0.451 | 0.343 to 0.584 | <0.001 |
| Difficulty | Elementary | FKGL | 38 | 0.249 | 0.198 to 0.343 | <0.001 |
| Difficulty | Intermediate | Expert-rated factual accuracy | 50 | 0.032 | 0.021 to 0.094 | 0.130 |
| Difficulty | Intermediate | Clinical Reasoning | 50 | 0.257 | 0.199 to 0.347 | <0.001 |
| Difficulty | Intermediate | GQS | 50 | 0.254 | 0.181 to 0.362 | <0.001 |
| Difficulty | Intermediate | FKGL | 50 | 0.293 | 0.238 to 0.378 | <0.001 |
| Difficulty | Advanced | Expert-rated factual accuracy | 51 | 0.059 | 0.035 to 0.135 | 0.003 |
| Difficulty | Advanced | Clinical Reasoning | 51 | 0.278 | 0.224 to 0.365 | <0.001 |
| Difficulty | Advanced | GQS | 51 | 0.339 | 0.272 to 0.446 | <0.001 |
| Difficulty | Advanced | FKGL | 51 | 0.192 | 0.156 to 0.274 | <0.001 |
| Difficulty | Expert | Expert-rated factual accuracy | 61 | 0.050 | 0.026 to 0.113 | 0.003 |
| Difficulty | Expert | Clinical Reasoning | 61 | 0.234 | 0.184 to 0.313 | <0.001 |
| Difficulty | Expert | GQS | 61 | 0.259 | 0.202 to 0.335 | <0.001 |
| Difficulty | Expert | FKGL | 61 | 0.280 | 0.235 to 0.352 | <0.001 |

## Supplementary Table S5. Safety & Ethics model-specific performance

| **Model** | **Expert-rated factual accuracy** | **Clinical reasoning (95% CI)** | **GQS (95% CI)** | **FKGL** |
| --- | --- | --- | --- | --- |
| Qwen3.6-plus | 3.90 | 4.78 (4.65-4.90) | 4.80 (4.67-4.92) | 23.81 |
| gpt-5.5 | 4.03 | 4.72 (4.53-4.90) | 4.72 (4.50-4.90) | 18.87 |
| Kimi-K2.6 | 3.90 | 4.55 (4.35-4.72) | 4.62 (4.45-4.78) | 20.22 |
| Qwen3.6-flash | 3.88 | 4.50 (4.28-4.72) | 4.60 (4.40-4.80) | 24.35 |
| GLM-5.1 | 3.88 | 4.40 (4.22-4.58) | 4.40 (4.22-4.58) | 19.40 |
| deepseek-v4-pro | 3.92 | 4.22 (4.08-4.38) | 4.10 (3.90-4.30) | 21.27 |
| Doubao-Seed-2.0-pro | 3.88 | 4.15 (4.03-4.30) | 4.03 (3.85-4.20) | 21.81 |
| deepseek-v4-flash | 3.92 | 4.00 (3.85-4.15) | 3.88 (3.70-4.03) | 20.19 |
| Doubao-Seed-2.0-lite | 3.85 | 4.03 (3.85-4.20) | 3.83 (3.62-4.00) | 21.23 |

## Supplementary Table S6. Response-length sensitivity analysis

| **Outcome** | **Standardized beta** | **95% CI** | **P** |
| --- | --- | --- | --- |
| Expert-rated factual accuracy | 0.055 | -0.069 to 0.179 | 0.388 |
| Clinical Reasoning | 0.554 | 0.441 to 0.668 | <0.001 |
| GQS | 0.683 | 0.560 to 0.806 | <0.001 |
| FKGL | -0.015 | -0.122 to 0.092 | 0.784 |

**Supplementary Table S7. Representative error patterns identified in large language model responses**

| **Item** | **Question focus / reference** | **Representative model error** | **Error category** | **Interpretation** |
| --- | --- | --- | --- | --- |
| PD-0063 True/False; Diagnosis and Differential Diagnosis; Advanced | One supportive criterion vs two red flags under the MDS diagnostic criteria. Reference: False; supportive criteria must outnumber red flags. | A model selected True and treated the presence of one supportive criterion as sufficient despite more red flags than supportive criteria. | Incorrect application of diagnostic criteria | Relevant concepts were recognized, but the quantitative rule in the MDS criteria was applied incorrectly. |
| PD-0064 Single-choice; Diagnosis and Differential Diagnosis; Advanced | Prodromal marker with the strongest positive likelihood ratio. Reference: PSG-confirmed REM sleep behavior disorder. | A model selected another biomarker and overestimated its predictive value relative to PSG-confirmed RBD. | Misinterpretation of clinical evidence strength | The response identified relevant biomarkers but ranked their clinical significance incorrectly. |
| PD-0153 True/False; Clinical Reasoning; Expert | DBS management during acute ischemic stroke. Reference: True; special acute-phase DBS management and MRI-conditional safety checks are required. | A model answered No and argued that DBS should not routinely be turned off during the acute stroke phase. | Incorrect management recommendation | MRI-compatibility issues were recognized, but the prespecified acute-phase DBS management recommendation was not followed. |
| PD-0021 Single-choice; Pathogenesis; Advanced | Mechanism not involved in alpha-synuclein cell-to-cell propagation. Reference: leukocyte-mediated transport across the blood-brain barrier. | A model selected an incorrect option while accurately describing several established propagation mechanisms. | Factual error despite partial knowledge | The response demonstrated related mechanistic knowledge but failed to identify the incorrect mechanism. |
| PD-0073 Multiple-choice; Diagnosis and Differential Diagnosis; Expert | Risk markers in the MDS research criteria for prodromal PD. Reference: the complete prespecified option set. | A model selected only a subset of the listed markers and therefore produced an incomplete option set. | Conceptual classification / incomplete selection error | The response showed relevant knowledge but did not reproduce the complete prespecified marker set. |
