# Supplementary material for: Comparative performance of nine large language models on professional Parkinson’s disease tasks: a multidimensional benchmark study
Source: Front Neurol. 2026 Oct 30;17:1970459. doi: 10.3389/fneur.2026.1970459 (PMC13759406; doi:10.3389/fneur.2026.1970459)
Supplement: Supplementary file 2 [file Table_1.docx]

**Supplementary Table S1. Characteristics of the large language models evaluated in this study**

| **Model** | **Developer** | **API model designation / identifier** | **Access method** | **Provider API platform / URL** | **Account tier** | **Temperature** | **Thinking / reasoning settings** | **Web search / external retrieval** | **Model identity verification** | **Evaluation period** |
| --- | --- | --- | --- | --- | --- | --- | --- | --- | --- | --- |
| GPT-5.5 | OpenAI | GPT-5.5 | Official provider HTTP API via batch platform | OpenAI API (exact base URL not retained in supplied parameter record) | Standard account | 1.0 | Thinking enabled; exact provider-specific reasoning effort was not independently verified from the retained request record. | Disabled | API model identifier recorded by evaluation platform | July 20–27, 2026 |
| Kimi-K2.6 | Moonshot AI | Kimi-K2.6 | Official provider HTTP API via batch platform | Kimi API platform: https://platform.kimi.com/ | Standard account | 1.0 | Thinking enabled; exact provider-specific reasoning effort was not independently verified from the retained request record. | Disabled | API model identifier recorded by evaluation platform | July 20–27, 2026 |
| Qwen3.6-plus | Alibaba Cloud / Qwen | Qwen3.6-plus | Official provider HTTP API via batch platform | Alibaba Cloud Bailian API platform: https://bailian.console.aliyun.com/ | Standard account | 1.0 | Thinking enabled; exact provider-specific reasoning effort was not independently verified from the retained request record. | Disabled | API model identifier recorded by evaluation platform | July 20–27, 2026 |
| GLM-5.1 | Zhipu AI / Z.ai | GLM-5.1 | Official provider HTTP API via batch platform | BigModel API platform: https://open.bigmodel.cn/ | Standard account | 1.0 | Thinking enabled; exact provider-specific reasoning effort was not independently verified from the retained request record. | Disabled | API model identifier recorded by evaluation platform | July 20–27, 2026 |
| Qwen3.6-flash | Alibaba Cloud / Qwen | Qwen3.6-flash | Official provider HTTP API via batch platform | Alibaba Cloud Bailian API platform: https://bailian.console.aliyun.com/ | Standard account | 1.0 | Thinking enabled; exact provider-specific reasoning effort was not independently verified from the retained request record. | Disabled | API model identifier recorded by evaluation platform | July 20–27, 2026 |
| DeepSeek-v4-pro | DeepSeek | DeepSeek-v4-pro | Official provider HTTP API via batch platform | DeepSeek API (exact base URL not retained in supplied parameter record) | Standard account | 1.0 | Thinking enabled; exact provider-specific reasoning effort was not independently verified from the retained request record. | Disabled | API model identifier recorded by evaluation platform | July 20–27, 2026 |
| Doubao-Seed-2.0-pro | ByteDance Seed | Doubao-Seed-2.0-pro | Official provider HTTP API via batch platform | Volcengine API platform: https://console.volcengine.com/ | Standard account | 1.0 | Thinking enabled; exact provider-specific reasoning effort was not independently verified from the retained request record. | Disabled | API model identifier recorded by evaluation platform | July 20–27, 2026 |
| DeepSeek-v4-flash | DeepSeek | DeepSeek-v4-flash | Official provider HTTP API via batch platform | DeepSeek API (exact base URL not retained in supplied parameter record) | Standard account | 1.0 | Thinking enabled; exact provider-specific reasoning effort was not independently verified from the retained request record. | Disabled | API model identifier recorded by evaluation platform | July 20–27, 2026 |
| Doubao-Seed-2.0-lite | ByteDance Seed | Doubao-Seed-2.0-lite | Official provider HTTP API via batch platform | Volcengine API platform: https://console.volcengine.com/ | Standard account | 1.0 | Thinking enabled; exact provider-specific reasoning effort was not independently verified from the retained request record. | Disabled | API model identifier recorded by evaluation platform | July 20–27, 2026 |

**Note.** All models were evaluated programmatically through the corresponding providers’ official APIs using a standardized batch-evaluation workflow. Temperature was configured to 1.0 for all evaluated configurations. Thinking/reasoning mode was enabled where supported; model-specific reasoning settings are reported according to the retained request records, and settings that could not be independently verified are explicitly indicated in the table. Web search, browsing, retrieval-augmented generation, external retrieval, and auxiliary tool use were disabled during the primary evaluation. Model identity was verified using the API model designation/identifier specified in each request and, where available, corroborated by returned API metadata. Provider-side backend snapshots, routing behavior, and updates not exposed through the API could not be independently verified. No refusals, truncated responses, rate-limit failures, or other technical generation failures occurred during the primary evaluation.
