# Supplementary material for: Comparative performance of nine large language models on professional Parkinson’s disease tasks: a multidimensional benchmark study
Source: Front Neurol. 2026 Oct 30;17:1970459. doi: 10.3389/fneur.2026.1970459 (PMC13759406; doi:10.3389/fneur.2026.1970459)
Supplement: Supplementary file 3 [file Table_2.docx]

**Supplementary Table S2. Operational scoring criteria for manual evaluation of LLM responses**

| **Dimension** | **Score** | **Operational criteria** |
| --- | --- | --- |
| **Expert-rated factual accuracy** | **1** | Completely incorrect or misleading. The final answer is wrong and/or the explanation contains major factual errors that substantially conflict with the reference answer or established clinical knowledge. |
|  | **2** | Mostly incorrect. Some correct information is present, but major factual errors, incorrect conclusions, or important misconceptions remain. |
|  | **3** | Partially correct. The core answer is broadly acceptable, but one or more clinically relevant inaccuracies, ambiguities, or substantive omissions limit factual correctness. |
|  | **4** | Mostly correct. The final answer and explanation are accurate overall, with only minor factual imprecision or nonessential omissions that do not alter the main conclusion. |
|  | **5** | Fully correct. The final answer and accompanying explanation are factually accurate, consistent with the reference answer, and free of clinically meaningful errors. |
| **Clinical Reasoning** | **1** | Reasoning is absent, incoherent, or clinically inappropriate. The response does not connect relevant information to the conclusion or relies on clearly flawed logic. |
|  | **2** | Reasoning is limited or substantially flawed. Some relevant considerations are mentioned, but important clinical relationships are missed, misinterpreted, or poorly integrated. |
|  | **3** | Reasoning is generally plausible but incomplete. The response identifies several relevant considerations but shows gaps in integration, prioritization, differential reasoning, or risk-benefit assessment. |
|  | **4** | Reasoning is coherent and clinically appropriate. Relevant information is integrated logically, with only minor omissions or limited depth in justification or qualification. |
|  | **5** | Reasoning is comprehensive, coherent, and clinically well integrated. The response appropriately weighs relevant findings, alternatives, risks, and uncertainty and provides a well-justified conclusion. |
| **Global Quality Scale (GQS)** | **1** | Very poor overall quality. The response is substantially incomplete, unclear, poorly organized, or of little practical value for the professional question. |
|  | **2** | Poor overall quality. Some useful information is provided, but important content is missing or the response lacks sufficient clarity, organization, or usefulness. |
|  | **3** | Moderate overall quality. The response is generally understandable and provides useful information, but completeness, clarity, organization, or practical usefulness is inconsistent. |
|  | **4** | Good overall quality. The response is clear, well organized, and sufficiently complete for the question, with only minor limitations in detail, clarity, or usefulness. |
|  | **5** | Excellent overall quality. The response is clear, well organized, comprehensive, appropriately focused, and highly useful for addressing the professional question. |
